# 

**Published:** 1885-02

**Authors:** 


SUBSCRIPTION PRICE, $3.00 PER YEAR.

Whole Number, 302. FEBRUARY, 1885. Vol. L., Number 2.

LIBRARY
INDEXED
S.G.O.

### THE CHICAGO MEDICAL JOURNAL AND EXAMINER

EDITORS:

S. J. JONES, M.D., L.L.D.

W. W. JAGGARD, A.M., M.D. HAROLD N. MOYER, M.D.

CHICAGO:

PUBLISHED BY THE CHICAGO MEDICAL PRESS ASSOCIATION,

240 WABASH AVENUE.

CLARK & LONGLEY, PRINTERS, 163 AND 165 DEARBORN STREET, CHICAGO.

